# Exposure to Insecticides Reduces Populations of *Rhynchophorus palmarum* in Oil Palm Plantations with Bud Rot Disease

**DOI:** 10.3390/insects10040111

**Published:** 2019-04-19

**Authors:** Luis Carlos Martínez, Angelica Plata-Rueda, Francisco Andrés Rodríguez-Dimaté, Juliana Mendonça Campos, Valdeir Celestino dos Santos Júnior, Gabriela Da Silva Rolim, Flavio Lemes Fernandes, Wiane Meloni Silva, Carlos Frederico Wilcken, José Cola Zanuncio, José Eduardo Serrão

**Affiliations:** 1 Departamento de Biologia Geral, Universidade Federal de Viçosa, Viçosa 36570-000, Minas Gerais, Brazil; jeserrao@ufv.br; 2 Instituto de Ciências Agrarias, Universidade Federal de Viçosa, Rio Paranaíba 38810-000, Minas Gerais, Brazil; angelicaplata@yahoo.com.mx (A.P.-R.); flaviofernandes@ufv.br (F.L.F.); 3 Departamento de Entomologia, Universidade Federal de Viçosa, Viçosa 36570-000, Minas Gerais, Brazil; ingpachogro@hotmail.com (F.A.R.-D.); scvaldeir@gmail.com (V.C.d.S.J.); zanuncio@ufv.br (J.C.Z.); 4 Departamento de Fitotecnia, Universidade Federal de Viçosa, Viçosa 36570-000, Minas Gerais, Brazil; mendonca.campos@yahoo.com.br (J.M.C.); gabrielasrolim@gmail.com (G.D.S.R.); 5 Departamento de Ciência Florestal, Universidade Federal de Viçosa, Viçosa 36570-000, Minas Gerais, Brazil; wianems@yahoo.com.br; 6 Departamento de Proteção de Plantas, Universidade Estadual Paulista, Botucatu 18603-970, São Paulo, Brazil; cwilcken@fca.unesp.br

**Keywords:** effects on reproduction, insect pest–disease association, insecticide efficacy, neurotoxicity, pest control, survivorship

## Abstract

The South American palm weevil (SAPW), *Rhynchophorus palmarum* Linnaeus (Coleoptera: Curculionidae) is the main pest of *Elaeis guineensis* and damages palm trees with bud rot disease in the Americas. The effects of six neurotoxic insecticides (abamectin, carbaryl, deltamethrin, fipronil, imidacloprid and spinosad) were evaluated against SAPW for toxicity, survival, reproduction, and mortality. Abamectin (LC_50_ = 0.33 mg mL^−1^), Carbaryl (LC_50_ = 0.24 mg mL^−1^), deltamethrin (LC_50_ = 0.17 mg mL^−1^), and fipronil (LC_50_ = 0.42 mg mL^−1^) were the most toxic to SAPW. Adult survival was 95% without exposure to insecticides, decreasing to 78–65% in insects treated with the LC_25_ and 49–35% in insects exposed to LC_50_. Sublethal doses of carbaryl, fipronil and imidacloprid showed significant effect on the reproduction of this insect. Mortality of SAPW populations caused by insecticides had similar effects in the laboratory and field conditions. The results suggest that carbaryl, deltamethrin, fipronil, and imidacloprid caused significantly higher mortality as compared to the control in SAPW and may be used to control its populations in oil palm trees where bud rot appears as the key disease for SAPW attraction and infestation.

## 1. Introduction

The South American palm weevil (SAPW), *Rhynchophorus palmarum* Linnaeus (Coleoptera: Curculionidae) is the most destructive insect of oil palm (*Elaeis guineensis* Jacquin) in the Americas. The SAPW also damages other palm tree species as *Attalea maripa* (Aubl. Mart.), *Cocos nucifera* (L.), *Elaeis oleifera* (Kunth), *Jessenia bataua* (Mart.), *Mauritia flexuosa* (L.F.), and *Phoenix sylvestris* (L.) [1,2]. This insect has bimodal activity habits, with pest activity peaks at 07:00 to 11:00 h and 17:00 to 19:00 h [3,4]. The life cycle of the SAPW is 164 days (egg 2–5, larva 45–70, pupa 25–45, and adult 60–85) [1,3]. In Colombia, SAPW can reach high infestations in palms during different steps of the Bud Rot disease (*Phytophthora palmivora* E.J. Butler) progression [5].

The causal agent of bud rot disease, *P. palmivora*, is widespread in oil palm plantations in the Americas. The sporangia develop in the meristem of the palm trees, causing necrotic lesions in the younger leaves which cause those leaves to rot and fall, thus reducing foliar area, fruit quality and oil production [6,7]. In addition, leaves decomposing in the oil palm canopy produce volatile compounds through fermentation that attracts SAPW adults [8]. Females of the SAPW drill into the rotting or juicy leaf, where they lay their eggs, and the hatching larvae begin feeding before moving to the plant meristem [1,5]. Oil palm can stop growing and plant death occurs because the meristem is consumed by larvae [1,5] and this tissue is not recoverable in monocots such as *E. guineensis*.

The SAPW–bud rot disease association causes death and total loss of palm trees [5]. This association has destroyed entire oil palm plantations in several countries including Brazil, Colombia, Ecuador, and Suriname [6,7,8]. In this context, the combined impact of the SAPW and bud rot disease represents a phytosanitary emergency in the Americas due to high infestation of oil palm plantations. Several cases of other insect pest–disease associations were reported in *E. guineensis* with *Acharia fusca* Stoll (Lepidoptera: Limacodidae) and *Stenoma impressella* Busck (Lepidoptera: Elachistidae) with *Pestalotiopsis* fungal complex [9,10,11], *Haplaxius crudus* Van Duzee (Hemiptera: Cixiidae) with fatal yellowing [12], and SAPW with the red-ring nematode disease caused by *Bursaphelenchus cocophilus* Cobb (Parasitaphelenchidae) [13].

The capture of SAPW adults with traps using the aggregation pheromone (*E*-6-Methyl-2-hepten-4-ol) is the main control method for this insect in oil palm plantations [14]. Massive traps pheromone system is efficient in crops but may not protect palm trees with bud rot disease from SAPW infestations [6]. Biological control with the parasitoids *Billaea rhynchophorae* Blanchard and *Paratheresia menezesi* Townsend (Diptera: Tachinidae) have potential for managing SAPW, but mass rearing methods for these insects are still in development [15,16].

Application of chemical insecticides is an efficient method for managing oil palm pest populations [17,18]. Other palm tree pests such as *Rhynchophorus cruentatus* Fabricius and *Rhynchophorus ferrugineus* Oliver (Coleoptera: Curculionidae) have been controlled with insecticides as an effective alternative [19,20]. Active neurotoxic ingredients such as abamectin, carbaryl, deltamethrin, fipronil, imidacloprid, and spinosad have different modes of action and are registered with the environmental regulatory agency United States, the Environmental Protection Agency (EPA) [21,22]. Abamectin affects the neuronal membrane of insects and acts as an antagonist to the γ–aminobutyric acid (GABA)–gated chloride channel [23,24,25]. Carbaryl inhibits acetylcholinesterase and thereby leads to the accumulation of the neurotransmitter acetylcholine, increasing stimulation of the cholinergic postsynaptic receptors [26,27,28]. Deltamethrin acts in the axonal membranes and prevent the closure of the voltage-gated sodium channels [29,30,31]. Fipronil disrupts blocking the GABA chloride channel and glutamate-gated chloride (GluCl) channels [31,32,33,34]. Imidacloprid interferes with the transmission of nerve impulses in insects by irreversible and specific binding to nicotinic acetylcholine receptors [35,36,37]. Spinosad is neurotoxic and it binds to the specific nicotinic acetylcholine and GABA receptors [38,39,40]. These novel insecticides can be used against the oil palm pest because of their suitability for integrated pest management (IPM) programs.

In this study, we evaluated the toxicity, survivorship, and impact on reproduction caused by neurotoxic insecticides on the SAPW in order to contribute to the development of strategies for controlling this insect pest, mainly in areas where bud rot disease appears as the key for SAPW attraction and infestation.

## 2. Materials and Methods

### 2.1. Insects

In the field, 347 SAPW adults (♂ = 128, ♀ = 219) were collected with traps in 7-year old commercial oil palm plantations in the county of Puerto Wilches, State of Santander, Colombia (07°25′ N, 73°57′ W). Traps were made of plastic containers (20 L) with two side holes on the top (8 × 12 cm). A mixture of vegetable substrate (250 g of sugarcane + 250 mL water in the ratio 1:1) and 5 mL of aggregation pheromone (*E*-6-Methyl-2-hepten-4-ol) obtained from the Chemical Products Laboratory of the National Institute of Agricultural Research (INRA, Versailles, France) was placed in each trap in a polystyrene bag. The captured insects were placed in plastic boxes (50 × 50 × 70 cm) with a perforated lid for ventilation and were transported to the agronomy laboratory of the University of Peace (UNIPAZ, Barrancabermeja, Santander, Colombia) to establish a mass rearing in the laboratory. Males and females of the SAPW were isolated in plastic trays (60 cm long × 40 cm wide × 30 cm high) which contained *E. guineensis* meristem. To collect the eggs, 900 oviposited eggs on the surface of the meristem were collected every 24 h and placed in Petri dishes (90 × 15 mm) containing a cotton saturated with distilled water. After hatching, first-instar larvae (*n* = 700) were placed individually in plastic boxes (10 × 20 cm) covered with cotton and fed every 24 h with sugarcane [1]. Larvae and pupae were maintained in an incubator (28 ± 2 °C, 75–85% RH, and a photoperiod of 12 h [Light:Dark]) until adult emergence. Emerged adults were placed in plastic trays (60 cm long × 40 cm wide × 30 cm high) and fed on *E. guineensis* meristem. SAPW adults were kept in the laboratory (28 ± 2 °C, 75–85% RH, and a photoperiod of 12 h [L:D]). Healthy SAPW adults with 72 h-old (emerged after to escape of the pupal cell) were used in the bioassays.

### 2.2. Concentration-Mortality Bioassay

Neurotoxic insecticides with different action modes and insecticidal contact activities (according to the commercial formulations) were used in all bioassays. The following insecticides abamectin (Vertimec^®^, Syngenta, Basel, Swaziland), 18 g L^−1^; carbaryl (Carbaril^®^, Drexel Chemical, Memphis, TN, USA), 480 g L^−1^; deltamethrin (Decis^®^ Bayer, Leverkusen, North Rhine-Westphalia, Germany), 25 g L^−1^; fipronil (Regent^®^, Bayer, Leverkusen, North Rhine-Westphalia, Germany), 200 g L^−1^; imidacloprid (Confidor^®^, Cyanamid, Wayne, NJ, USA), 200 g L^−1^; and spinosad (Tracer^®^, Dow Agrosciences, Zionsville, IN, USA), 120 g L^−1^ were diluted in 1 L of distilled water to obtain the stock solutions. After 30 min of stock solution preparation, six concentrations of each insecticide were then prepared and used to assess the insecticide toxicity and determine relevant toxicological endpoints; a dilution series of concentrations (0.062, 0.125, 0.25, 0.5, 1 mg mL^−1^, and 2 mg mL^−1^) was used to determine the concentration–mortality relationship and lethal concentrations (LC_25_, LC_50_, LC_75_, and LC_90_). Distilled water was used as a control. Subsequently, each insecticide solution (10 µL) was applied to the thorax of 100 SAPW adults (males/females, 1 ratio) using a micropipette (Eppendorf^®^ 1–10 µL, Hamburg, Deutschland). The exposed insects were placed individually in polystyrene boxes (12 × 15 cm) and maintained in a photoperiod of 12 h [L:D]. Oil palm meristem blocks were provided as food and sectioned 1 h before insecticide exposure. Three replicates with 100 adults each were used for each of the six concentrations tested, following a completely random design. Mortality was registered after insecticide exposure over 2 d.

### 2.3. Time-Mortality Bioassay

The time-mortality bioassays for the SAPW using the insecticide concentrations obtained from the concentration-mortality bioassay were carried out to determine the lethal toxicity. Adults of the SAPW were exposed to LC_25_ and LC_50_ of each insecticide, as determined in the toxicity bioassay, mortality was recorded after every 12 d. Exposure procedures, conditions, and number of insects were the same as those described above for the toxicity test. Three replications with 50 adults each were used to verify the insecticide concentrations, following a completely random design.

### 2.4. Insecticide Effects on Reproduction

Adults of the SAPW were isolated in plastic containers (25 × 25 × 25 cm), containing *E. guineensis* meristem. The calculated lethal concentration (LC_25_) of each insecticide was topically applied to the thorax of the insects (males/females, 1 ratio). Distilled water was used as a control. A total twenty-five pairs of SAPW adults were individually evaluated every day until the female died. Each adult pair was checked daily and the eggs were counted, collected, and transferred into new glass containers. Meristem on which the adults had been feeding was then inspected and eggs were collected per day. Then, copulation (mated females) was recorded and females that had not produced eggs during longevity were considered to have failed to mate. Also, fecundity (average number of eggs produced/mated females) and viability (eggs hatched)/total eggs (hatched + unhatched eggs) was calculated. The number of offspring/females was calculated as: percentage of copulation × fecundity × viability.

### 2.5. Field Assays in Palm Trees with Bud Rot Disease

The bioassay was conducted in 7-year-old commercial oil palm plantations (cv ‘Tenera’ × ‘Deli Ghana’) in the county of Puerto Wilches (Santander, Colombia), with an average temperature of 28.46 °C, 75–92% relative humidity, 1580–2155 h annual sunshine, and 2283 mm annual rainfall. In these natural conditions, 420 palm trees with early symptoms of bud rot disease were selected [41] where high infestations of the SAPW have been found in previous studies [5]. Adults of this insect (males/females, 1 ratio) were used for each treatment in the controlled field test. For each palm tree, 50 adults were placed on the canopy, above the meristem and isolated with a nylon cage (0.5 × 0.5 × 1.20 m) for 15 d to ensure the different developmental stages of SAPW. Each insecticide at the calculated lethal concentration (LC_90_) as well as the control (distilled water) was used as treatment with four replications. Treatments were applied at the day 15th day after setting the cage, where adults had a reproductive period and subsequently, populations were obtained at different life stages. Applications of 1 L of insecticide solution per canopy were made by a hand sprayer (Sampoorti Agrocare^®^, New Delhi, India, 16 L capacity). The palm trees were cut, the trunk carefully dissected with a chainsaw (MS 880 Stihl Magnum^®^, Orlando, FL, USA), and checked with a magnifying glass for the presence of the SAPW in the stages of larva, pupa, and adult alive and dead, which were counted. For group of palms cut each 15 d, mortality of the SAPW caused by insecticides was recorded each 15 d during two months with an experimental design in randomized blocks.

### 2.6. Statistical Analysis

Concentration-mortality data were subjected to Probit analysis, generating concentration-mortality curve [42]. Time-mortality bioassay data were submitted to survival analysis using the Kaplan–Meier estimator (log-rank method) with the Origin Pro v 9.1 program [43]. The number of surviving SAPW adults at the end of the experiment was treated as censored data. Insecticidal effects on reproduction (percentage of copulation, fecundity, viability, and offspring/female) were analyzed by one-way ANOVA. For mortality in field conditions, a Kolmogorov-Smirnov test verified the data normality to meet the normality assumptions and ANOVA was conducted as a mixed model. A Tukey’s Honestly Significant Difference (HSD) test was also used for comparison of means at the 5% significance level. Mortality data were summarized in percentages and all values presented as mean ± SEM. Concentration-mortality, reproduction, and mortality data in field conditions were analyzed using SAS User software (v. 9.0) for Windows [44].

## 3. Results

### 3.1. Toxicity

The concentration–mortality model used was suitable (χ^2^; *p* < 0.001) confirming the toxicity of each insecticide to SAPW and allowing the estimates of the desired toxicological endpoints for subsequent use (Table 1). For the LC_50_ estimated value, bioassay indicated that deltamethrin with LC_50_ = 0.17 (0.03–0.29) mg mL^−1^ and carbaryl with LC_50_ = 0.24 (0.08–0.33) mg mL^−1^ were the most toxic insecticides to the SAPW adults followed by abamectin with LC_50_ = 0.33 (0.26–0.61) mg mL^−1^, fipronil with LC_50_ = 0.42 (0.40–0.44) mg mL^−1^, spinosad with LC_50_ = 0.54 (0.19–0.62) mg mL^−1^, and imidacloprid with LC_50_ = 0.66 (0.56–0.84) mg mL^−1^. Mortality remained < 1% in the control.

### 3.2. Survival Analysis

**Figure 1:**
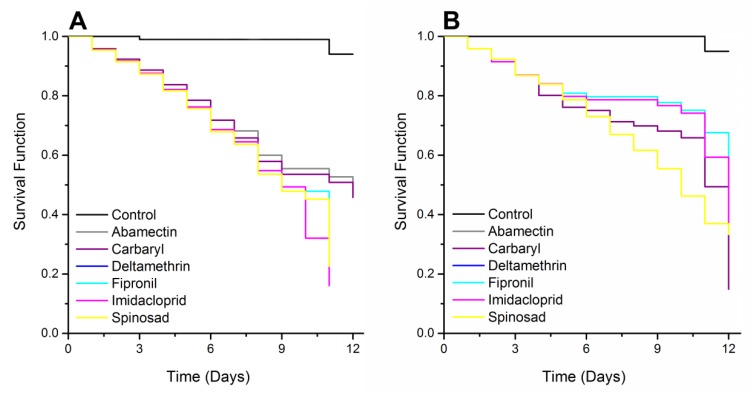
Survival curves of *Rhynchophorus palmarum* adults exposed to insecticides subjected to survival analyses using the Kaplan–Meier estimators’ log-rank test. Lethal concentration (**A**) LC_50_ (χ^2^ = 59.88; *p* < 0.001) and (**B**) LC_25_ (χ^2^ = 51.03; *p* < 0.001).

The survivorship was recorded when the pest was exposed from 12 d to the various insecticides each applied at different lethal concentrations (Figure 1). The survival analysis of the data from the SAPW adults exposed to lethal concentration LC_50_ of each insecticide indicated differences (log-rank test, χ^2^ = 59.88, df = 6, *p* < 0.001). Survivorship was 95.1% in the control, decreasing to 47.4% with abamectin, 45.7% with carbaryl, 35.8% with deltamethrin, 41.8% with fipronil, 49.3% with imidacloprid, and 45.1% with spinosad. Survivorship of the SAPW exposed to lethal concentration LC_25_ of each insecticide showed differences (log-rank test, χ^2^ = 51.03, df = 6, *p* < 0.001). Survivorship was 96.8% in the control, decreasing to 74.1% with abamectin, 65.8% with carbaryl, 76.7% with deltamethrin, 76.1% with fipronil, 78.6% with imidacloprid, and 78.3% with spinosad.

### 3.3. Insecticide Effects on Reproduction

**Figure 2:**
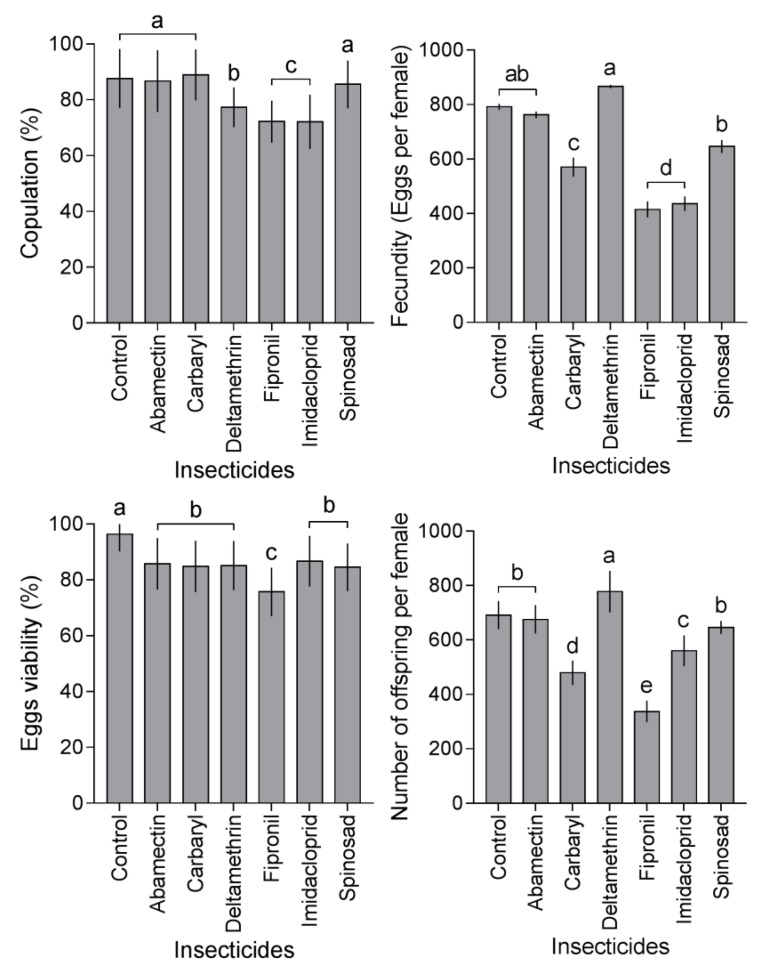
Effects on the reproduction of *Rhynchophorus palmarum* caused by sublethal concentration LC_25_ of the six insecticides: percentage of copulation, fecundity, eggs viability and offspring/female (Mean ± SEM). In each graph, values in the same column with different letters show significant differences by Tukey’s HSD test at the *p* < 0.05 level.

The effects caused by six insecticides on reproductive factors of the SAPW, such as percentage of copulation, fecundity, eggs viability and number of offsprings per females, were determined (Figure 2). The percentage of copulation of the SAPW was different between insecticides tested with concentrations estimated for the LC_25_ values (F_6,24_ = 9.46, *p* < 0.05). The percentage of copulation was high when the insects were exposed to carbaryl (88.9%), control group (87.6%), abamectin (86.7%), and spinosad (85.5%) and less with deltamethrin (77.3%), fipronil (72.2%), and imidacloprid (72.1%). The fecundity differed between the insecticides tested (F_6,24_ = 11.73, *p* < 0.05). The number of eggs per female was high with deltamethrin (886 ± 6.2), abamectin (791 ± 11), and the control group (762 ± 12) and significantly lower with imidacloprid (436 ± 26) and fipronil (414 ± 29). The eggs viability was among different insecticides tested (F_6,24_ = 7.85, *p* < 0.05). Eggs viability was high in the control group (96.3%), following by imidacloprid (86.7%), abamectin (85.7%), deltamethrin (85.1%), carbaryl (84.4%), and spinosad (84.5%). Insecticides reduced the SAPW offspring per female (F_6,24_ = 11.52, *p* < 0.05), being high in deltamethrin (777 ± 76), control group (691 ± 51), abamectin (675 ± 52), and spinosad (646 ± 24).

### 3.4. Mortality in Field Conditions

**Figure 3:**
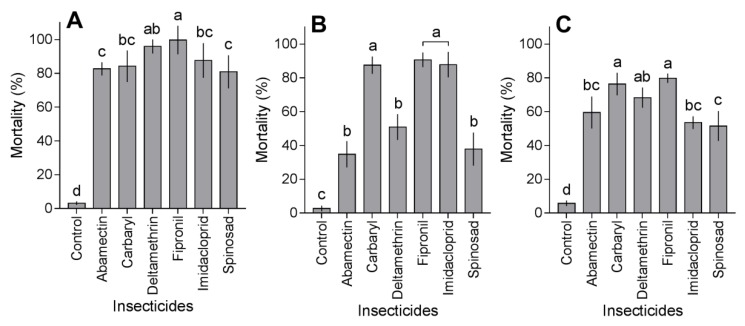
Mortality of *Rhynchophorus palmarum* by insecticides to level LC_90_ application on palm trees with bud rot disease: larvae (**A**), pupae (**B**) and adults (**C**). Treatments means (percent mortality ± SEM) with different letters show significant differences by Tukey’s HSD test at the *p* < 0.05 level.

The mortality effects caused by the tested insecticides on the SAPW larvae were different in the field conditions, using the previously estimated concentrations for the LC_90_ values (F_6,139_ = 76.29; *p* < 0.05). Fipronil and deltamethrin caused mortalities of 99.6 ± 0.2% and 95.9 ± 2.1%, respectively, followed by mortalities by imidacloprid, carbaryl, abamectin, and spinosad of 87.5 ± 5.1%, 84.2 ± 4.6%, 82.6 ± 1.9%, and 80.9 ± 4.9%, respectively. Mortality never exceeded 3.16 ± 1.1% in the control (Figure 3A). Mortality of the SAPW pupae differed between insecticides in the field according to the LC_90_ values (F_6,139_ = 53,06; *p* < 0.05). Fipronil, imidacloprid, and carbaryl caused mortality rates of 90.6 ± 5.9%, 87.8 ± 4.3%, and 87.5 ± 5.1%, respectively, while deltamethrin, abamectin and spinosad caused lower mortality of 50.8 ± 7.6%, 37.8% ± 7.5%, and 34.7 ± 7.7%, respectively. Mortality did not exceed 2.66 ± 1.6% in the control (Figure 3B). Mortality of the SAPW adults differed between insecticides according to the estimated LC_90_ (F_6,139_ = 35.18, *p* < 0.05). Fipronil, carbaryl, and deltamethrin caused mortality of 79.7 ± 1.4%, 76.3 ± 3.3%, and 68.2 ± 2.9%, respectively, followed by abamectin with 59.4 ± 9.4%. Imidacloprid and spinosad caused lower mortality with 53.5 ± 1.8% and 51.5 ± 4.4%, respectively. Mortality never exceeded 5.75 ± 0.8% in the control (Figure 3C).

## 4. Discussion

The use of various neurotoxic insecticides was effective in causing mortality, compromising survivorship, and reducing reproduction of the SAPW. These insecticides have the potential to be an effective component of an IPM program, mainly in oil palm plantations with bud rot disease.

The toxicity of six insecticides to the SAPW was determined from the bioassays performed under laboratory and field conditions. The insecticides deltamethrin, carbaryl, abamectin, fipronil, spinosad and imidacloprid were toxic to adult SAPWs and have a strong effect through topical application. The SAPW individuals exposed to high concentrations of each insecticide (LC_50_ and LC_90_) displayed altered locomotor activity. In some individuals, the constant paralysis at concentrations close to the LC_50_ and no sign of recovery show the effect of insecticides on the nervous system of the insect. In this study, the concentration–mortality bioassay indicated that evaluated concentrations of imidacloprid followed by spinosad were less toxic to the SAPW. However, insecticides such as abamectin, carbaryl, deltamethrin and fipronil can be used on the SAPW for their different modes of action and applied in rotation, thus avoiding the effects of insecticide resistance.

Survival analysis indicated that a significant proportion of variation in SAPW survival during our trials could be attributed to action mode differences among insecticides. However, extended periods of exposure to insecticides were necessary to induce mortality in the SAPW. In this study, the comparative effects on the SAPW between the neurotoxic insecticides were observed at various time points. The speed with which the insecticide acts on the insect is useful because can define the lethal effect quickly and consequently, essential to protect crops, especially when the target insect is a vector of dangerous pathogens as in the case of the SAPW [3,5,45]. Some insecticides cause cessation of feeding long before the target insects actually dies. However, a quick-acting insecticide can be essential for impregnation of the palm meristem; in this case, the weevils were able to feed, even if they died later. Neurotoxic insecticides and other toxic compounds can reduce injuries to oil palm by insect pests such as *Atta sexdens* Linnaeus (Hymenoptera: Formicidae) [46], *Demotispa neivai* Bondar (Coleoptera: Chrysomelidae) [47], *Leptopharsa gibbicarina* Froeschner (Hemiptera: Tingidae) [48], and *Strategus aloeus* Linnaeus [25].

Sublethal effects caused with LC_25_ of each insecticide on the reproduction of the SAPW were observed. Exposure to deltamethrin, fipronil, and imidacloprid affects the sexual behavior of male and female of this insect and causes a significant reduction in mating among pairs of virgin adults. In this case, adults disrupted mating for a long time period under laboratory conditions and some individuals treated were seen to recover the locomotor activity and their ability to mate. Various studies have reported the sexual behavior in insects after insecticide exposure, affecting the ability of male, sex pheromone signal detection, and sustained oriented flight [49,50,51]. With regard to fecundity, a smaller egg quantity was observed in females of the SAPW after carbaryl, fipronil, imidacloprid and spinosad exposure, although these females were stimulated (hormoligosis) to oviposit more eggs with deltamethrin. Effects on fecundity were studied in *Choristoneura fumiferana* Clemens (Lepidoptera: Tortricidae) exposed to carbaryl [52], *Helicoverpa armigera* Hübner (Lepidoptera: Noctuidae) exposed to spinosad [53], *Nephotettix virescens* Distant (Hemiptera Cicadellidae) exposed to imidacloprid [54], and *Plutella xylostella* Linnaeus (Lepidoptera: Plutellidae) exposed to fipronil [55], while hormoligosis was reported in *Nilaparvata lugens* Stål (Hemiptera: Delphacidae) exposed to pyrethroids [51]. Effects on egg viability were found and can be attributed to the different modes of action of these insecticides. In this context, insecticides cause abnormal egg hatching, disrupt hormonal balance, and alter embryonic developmental, compromising egg survival for various insects [46,56,57]. The results suggest that neurotoxic insecticides have high impact on the reproduction of the SAPW, affecting the olfactory response, fecundity, egg viability and offspring of this insect.

The lethal effect of the insecticides on the SAPW in palm trees with bud rot disease in the field was consistent with those observed in the laboratory. Fipronil, carbaryl, and deltamethrin showed lethal effects against the SAPW at different developmental stages. Abametic and spinosad were toxic for larva and adult, whereas imidacloprid was toxic for larva and pupa. However, mortality levels at the different developmental stages were lower than those obtained under laboratory conditions. It’s possible that the efficacy of insecticides in field conditions is due to contact exposure to the insect body [45] or by degradation during absorption and plant tissue translocation [58,59]. However, while it’s difficult to accurately know the amount of the insecticide absorbed by each insect, but mortality caused by these insecticides on the SAPW showed a similarity of that trend with the application of the lethal concentration (LC_90_). The lethal effect of insecticides and their effectiveness have also been studied in other Curculionidae pests under field conditions with fipronil being a potent control agent for *Rhynchophorus ferrugineus* Oliver [60], carbaryl for *Dendroctonus brevicomis* LeConte [61], and deltamethrin for *Sternochetus mangiferae* Fabricius [62]. Other studies show the efficiency of abamectin to control *Dendroctonus ponderosae* Hopkins [63], spinosad on *Sitophilus oryzae* Linnaeus [64], and imidacloprid for *Ips calligraphus* Germar [65]. The results have shown that each insecticide has a different spectrum of activity related to modes of action affecting the number of larvae, pupae, and adults of the SAPW. In particular, carbaryl, fipronil, and imidacloprid are the most effective in field and that maximum efficiency from insecticide treatments should be used during these life stages. The application of these insecticides reduces one or various developmental stages of the SAPW on palms affected by the bud rot disease, suggesting that continuous applications in the canopy of the palm tree can drastically decrease the population level of this insect.

## 5. Conclusions

The potential of the six neurotoxic insecticides for managing the SAPW was studied. The toxicity of these insecticides, each with different modes of action, may efficiently control SAPW populations and reduce the insect’s damage to oil palm trees with bud rot disease. Fipronil, carbaryl, deltamethrin and imidacloprid have lethal effects on larvae, pupae and adults of this insect with the potential to control its field populations. The results show that neurotoxic insecticides with different modes of action cause high mortality, reduce survivorship, and affect the insect reproduction. As such, these insecticides can be used in rotation to effectively manage SAPW populations.

## Figures and Tables

**Table 1:** Lethal concentrations of six insecticides on *Rhynchophorus palmarum* adults obtained from Probit analysis. For all insecticides, chi-square (χ^2^) for lethal concentrations and fiducial limits based on a log scale were statistically significant at the *p* < 0.001 level.

Insecticides	Lethal Concentration	Estimated Value (mg mL^−1^)	Confidence Interval (mg mL^−1^)	Slope (± SE)	χ^2^
Abamectin	25	0.22	0.06–0.39	1.21 (±0.11)	8.731
	50	0.33	0.26–0.51		
	75	0.43	0.19–1.57		
	90	0.52	0.35–2.96		
Carbaryl	25	0.17	0.06–0.19	3.15 (±2.23)	46.71
	50	0.24	0.08–0.33		
	75	0.41	0.31–0.52		
	90	0.56	0.46–0.74		
Deltamethrin	25	0.06	0.02–0.18	3.11 (±1.95)	21.28
	50	0.17	0.03–0.29		
	75	0.28	0.14–0.46		
	90	0.38	0.25–0.65		
Fipronil	25	0.35	0.32–0.37	3.94 (±1.49)	64.71
	50	0.42	0.40–0.44		
	75	0.50	0.48–0.52		
	90	0.57	0.55–0.60		
Imidacloprid	25	0.39	0.20–0.49	2.94 (±1.92)	34.61
	50	0.66	0.56–0.84		
	75	0.94	0.38–1.92		
	90	1.19	0.96–1.78		
Spinosad	25	0.26	0.15–0.37	1.37 (±0.19)	10.22
	50	0.54	0.19–0.62		
	75	0.91	0.78–1.04		
	90	1.35	1.21–1.53		
